# Peripheral Multiple Cytokine Profiles Identified CD39 as a Novel Biomarker for Diagnosis and Reflecting Disease Severity in Allergic Rhinitis Patients

**DOI:** 10.1155/2023/3217261

**Published:** 2023-05-10

**Authors:** Yuanwei Jiang, Weiqun Hu, Zhifu Cai, Chaofan Lin, Shengnan Ye

**Affiliations:** ^1^Department of Otolaryngology-Head and Neck Surgery, The First Affiliated Hospital of Fujian Medical University, Fuzhou, Fujian 350000, China; ^2^Department of Otolaryngology-Head and Neck Surgery, The Affiliated Hospital of Putian University, Putian, Fujian 351100, China

## Abstract

**Background:**

Allergic rhinitis (AR) is a common clinical problem, and immune cells and cytokines were proven to be pivotal in its pathogenesis. Our aim is to measure the peripheral concentrations of multiple cytokines in AR patients and identify novel biomarkers for diagnosis and disease severity.

**Methods:**

Peripheral blood samples were collected from 50 AR patients, including 25 mild AR (MAR) patients and 25 moderate-severe AR patients (MSAR), and 22 healthy controls (HCs), and multiple cytokine profiling was outlined by Luminex assay. Cytokine levels were compared among the three groups, and their correlations with disease severity were evaluated. The candidate cytokines were further verified by enzyme-linked immunosorbent assay (ELISA) in a validation cohort.

**Results:**

Multiple cytokine profiling revealed that CD39 and interferon (IFN)-*γ* levels were reduced, and interleukin (IL)-13, IL-5, IL-33, and thymic stromal lymphopoietin (TSLP) levels were elevated in the AR group than the HC group (*P* < 0.05). Receiver operating characteristic (ROC) curves presented that serum CD39 and IL-33 exhibited strong diagnostic abilities, and serum CD39 and IL-10 presented capacities in distinguishing disease severity (*AUC* > 0.8, *P* < 0.05). Moreover, CD39 concentrations were decreased, and IL-10, IL-5, and TSLP concentrations were enhanced in the MSAR group more than in the MAR group. Correlation analysis results showed that serum CD39, IL-5, and TSLP levels were associated with total nasal symptom score (TNSS) and visual analogue score (VAS) (*P* < 0.05). Further data in the validation cohort suggested that serum CD39 levels were reduced, and IL-5 and TSLP levels were increased in AR patients, especially in MSAR patients (*P* < 0.05). ROC results revealed potential values of serum CD39 in diagnosis and disease severity evaluation in AR patients (*P* < 0.05).

**Conclusion:**

This study highlighted that peripheral multiple cytokine profiles were significantly varied in AR patients and associated with disease severity. The results in discover–validation cohorts implied that serum CD39 might serve as a novel biomarker for diagnosing AR and reflecting its disease severity.

## 1. Introduction

Allergic rhinitis (AR) is a common chronic disease worldwide, and its prevalence continues to increase [1, 2]. Although multiple hypotheses have been proposed in terms of the etiologies of AR, its underlying pathogenesis remains ambiguous [2, 3]. In clinical practice, the AR diagnosis is conducted mostly by referring to the clinical history and laboratory findings, including skin tests and/or serum-specific IgE antibodies to allergens [4, 5]. Recent studies identified some novel biomarkers in diagnosing AR, including immune cells, cytokines, metabolites, and genes, which might contribute to a better understanding of its occurrence and development [6–9]. Accumulating evidence showed that AR was a highly heterogeneous disease with a wide degree of severity, which was not conducive to achieving individualized treatment [10, 11]. Although AR can be categorized into mild AR (MAR) and moderate to severe AR (MSAR) based on the self-rating symptom scales, such as total nasal symptom score (TNSS) and visual analogue score (VAS), no available objective indicator or biological marker was clinically applied in reflecting its disease severity and monitor its activities. Therefore, exploring disease-specific indicators in AR diagnosis and disease severity is extremely meaningful to improve patient management and benefit clinical translational research.

Previous studies demonstrated that immune cells and cytokines were crucial in the immunopathogenesis of AR, including T-helper cells, macrophages, B cells, and corresponding cytokines, and these potential candidates contributed to understanding ongoing pathophysiological changes and monitoring disease activities [12, 13]. Han et al. [7] found that serum IL-1*β* levels were elevated in the AR children, especially in severe persistent patients, and positively correlated with disease severity. A recent publication reported that several biomarkers were changed in nasal fluid-persistent AR patients, and Clara cell protein 16 (CC16) showed an inverse correlation with symptom scores [14]. However, relatively few studies have determined the multiple cytokine profiling in the peripheral blood of AR patients. Thus, the primary aim of this study was to outline peripheral multiple cytokine profiles in AR patients and explore novel biomarkers for diagnosis and disease severity by discovering and validating cohorts.

## 2. Methods

### 2.1. Subjects and Setting

We recruited 50 house dust mite (HDM)-induced AR patients, including 25 mild AR (MAR) patients and 25 moderate-severe AR patients (MSAR), and 22 healthy controls (HCs) at our department. AR was diagnosed by allergy specialists according to the allergic rhinitis and its impact on asthma (ARIA) guidelines. The inclusion criteria were listed as follows: positive reactions of skin prick tests to *Dermatophagoides farinae* (Der f) and/or *Dermatophagoides pteronyssinus* (Der p) (at least ++) and/or specific-IgE level against Der f or Der p (>0.35 IU/mL); age ≥ 18 and ≤60 years old. We excluded the following patients: those who refused to participate in this study; those who have taken antiallergic drugs or steroid consumption within 4 weeks before enrollment; with allergic fungal rhinitis, nasal, or sinuses carcinoma; with inflammatory or autoimmune diseases, severe liver, kidney dysfunction, and a history of immunotherapy. Serum samples were collected and preserved in our laboratory. Clinical variables include symptom scores, laboratory results, and demographic data. Twenty-two healthy controls were also enrolled as a control group in the present study. This study was approved by Ethics Committee in our hospital, and signed informed consent was provided by each participant.

### 2.2. Serum Sample Collection and Analysis of Serum Cytokines

Five millimeters of whole blood sample was collected from each participant and stored for 1 hour at room temperature. Blood samples were then centrifuged for 10 minutes at 3000 rpm, and the supernatant was harvested and stored at −80°C for later analysis. Individual serum samples were subjected to cytokine profile quantification by the Luminex system using a multiplex assay kit (BioRad, CA, USA). The kit protocol for quantitative analysis consisted of 21 total cytokines, including CD38, CD39, CD73, IFN (interferon) -*γ*, IL (interleukin)-10, IL-13, IL-17A, IL-1*β*, IL-2, IL-25, IL-3, IL-33, IL-4, IL-5, IL-6, IL-7, IL-8, IL-9, TGF-*β*1, TGF-*β*2, and TSLP. All experimental measurements were performed, referring to the kit manufacturer's instructions. The cytokine levels were analyzed with FCAP Array 3.0 software.

### 2.3. Validation Cohort and Potential Cytokine Validation

To further verify the potential biomarkers identified in the discovery cohort, another independent cohort consisting of 40 MSAR patients, 40 MAR patients, and 40 HCs was recruited, and whole blood samples were collected. The clinical and demographic data were collected. The potential cytokines concentrations were detected by commercial enzyme-linked immunosorbent assay (ELISA) kits (CUSABIO, Wuhan, China) according to the manufacturer's instructions.

### 2.4. Statistical Analysis

Statistical analysis was conducted with SPSS 23.0 (IBM, Armonk, NY, USA), and figures were constructed using GraphPad Prism 8.3 (GraphPad Software, San Diego, CA, USA). Numerical variables were displayed by mean ± standard deviation, and the Student's *t*-test and one-way analysis of variance (ANOVA) were applied between two groups and among three groups when the variables were normally distributed. Otherwise, Mann–Whitney U-test and Kruskal–Wallis H test were used when the data were not normally distributed. Categorical variable data were described as frequencies and percentages (%), and the Chi-square test was used for comparison. Spearman's correlation analysis was performed to evaluate the associations between cytokines and symptom scores. Receiver operating characteristic (ROC) curves were utilized to assess the abilities of potential cytokines in diagnosing AR and distinguishing its severity. A *P* < 0.05 is considered to be statistically significant.

## 3. Results

### 3.1. Baseline Characteristics of All Participants

Demographics and clinical data of 50 AR patients and 22 HCs were shown in Table 1. The rate of accompanying asthma was higher in the AR group than the HC group, and no statistical difference was observed in gender, age, BMI, and the rate of allergic conjunctivitis. In Table 2, TNSS and VAS were significantly lower in the MAR group than the MSAR group, but no statistical difference was observed in other variables between the two groups.

### 3.2. Serum Cytokine Profiles of AR Group vs. HC Group

**Figure 1:**
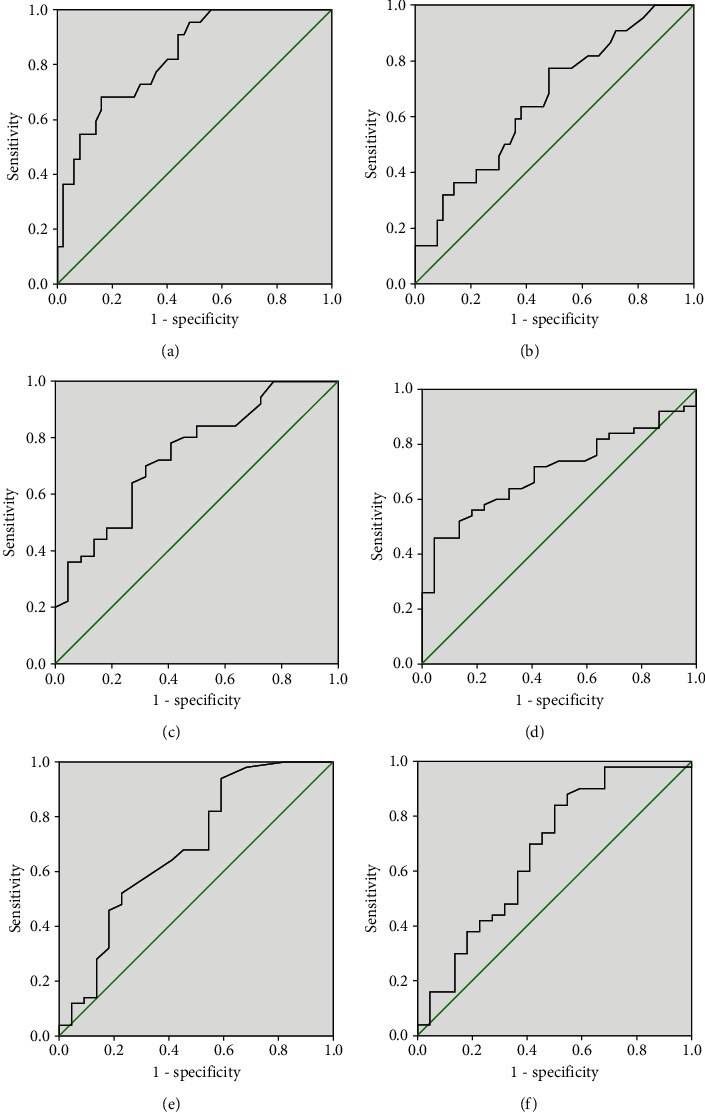
The ROC curves of potential cytokines for diagnosing AR. (a) CD39, (b) IFN-*γ*, (c) IL-13, (d) IL-33, (e) IL-5, and (f) TSLP. ROC: receiver operator characteristic; AR: allergic rhinitis; IFN: interferon alpha; IL: interleukin; TSLP: thymic stromal lymphopoietin.

**Figure 2:**
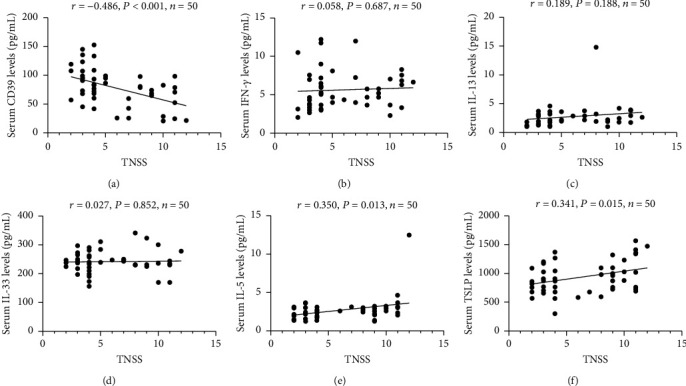
The associations between potential cytokines and TNSS in AR patients. TNSS: total nasal symptom score; AR: allergic rhinitis; IFN: interferon alpha; IL: interleukin; TSLP: thymic stromal lymphopoietin.

**Figure 3:**
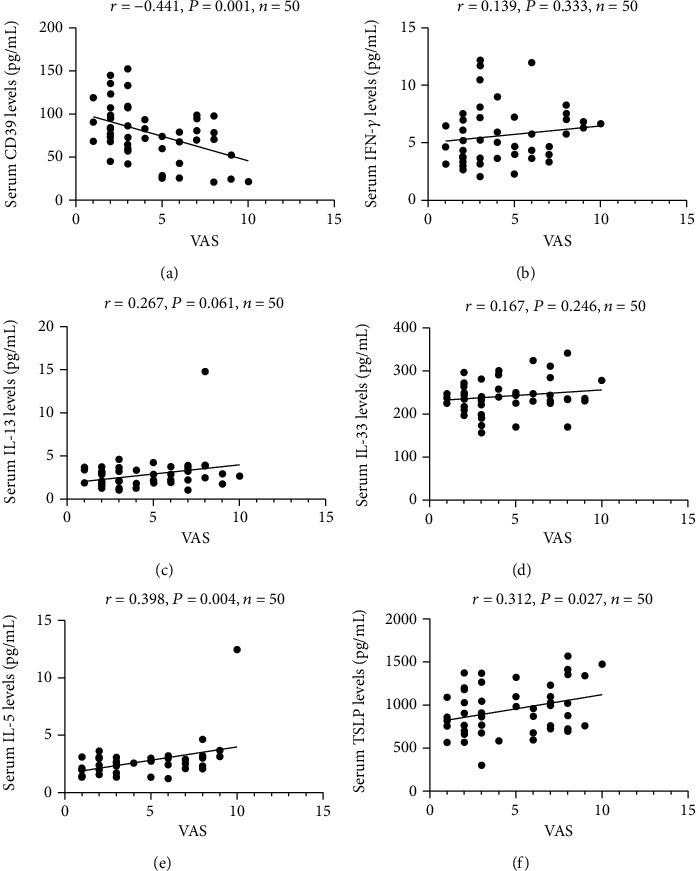
The associations between potential cytokines and VAS in AR patients. VAS: visual analogue score; AR: allergic rhinitis; IFN: interferon alpha; IL: interleukin; TSLP: thymic stromal lymphopoietin.

Table 3 presented the comparison of 21 cytokines levels between the AR group and the HC group. In the AR group, the serum concentrations of CD39 and IFN-*γ* were markedly decreased, and IL-13, IL-33, and TSLP were significantly increased than those in the HC group (*P* < 0.05). The ROC curves in Figure 1 showed that these 6 indicators exhibited different capabilities in diagnosing AR, and the detailed parameters are described in Table S1. Spearman's correlation analysis results in Figures 2 and 3 demonstrated that serum CD39 levels were inversely correlated with TNSS and VAS, and IL-5 and TSLP levels were associated with TNSS and VAS (*P* < 0.05).

### 3.3. Serum Cytokine Profiles of MAR Group vs. MSAR Group

**Figure 4:**
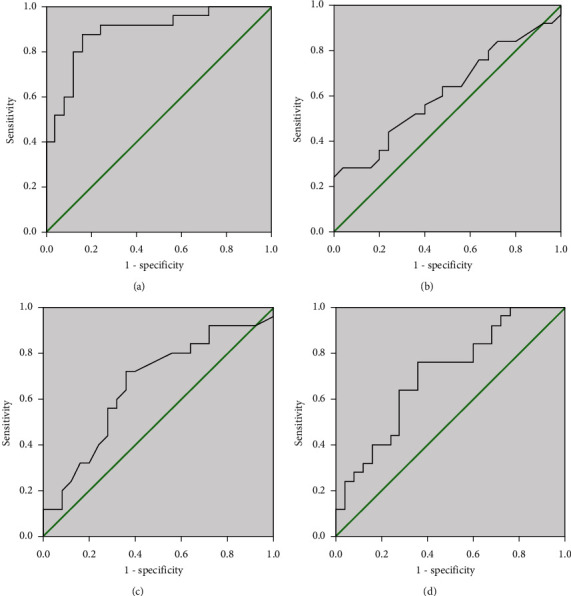
ROC curves of the capabilities of 4 cytokines in distinguishing MSAR from MAR. (a) CD39, (b) IL-10, (c) IL-5, and (d) TSLP. ROC: receiver operator characteristic; MAR: mild allergic rhinitis; MSAR: moderate-severe allergic rhinitis; IL: interleukin; TSLP: thymic stromal lymphopoietin.

The concentrations of 21 cytokines between the MAR group and MSAR group are listed in Table 4. The serum CD39 levels were reduced, and IL-10, IL-5, and TSLP levels were enhanced in the MSAR group than MAR group (*P* < 0.05). ROC curves suggested that CD39, IL-5, and TSLP presented potential abilities in distinguishing MSAR from MAR (*P* < 0.05, Figure 4), and the detailed variables were recorded in Table S2.

### 3.4. Potential Cytokine Verification in the Validation Cohort

**Figure 5:**
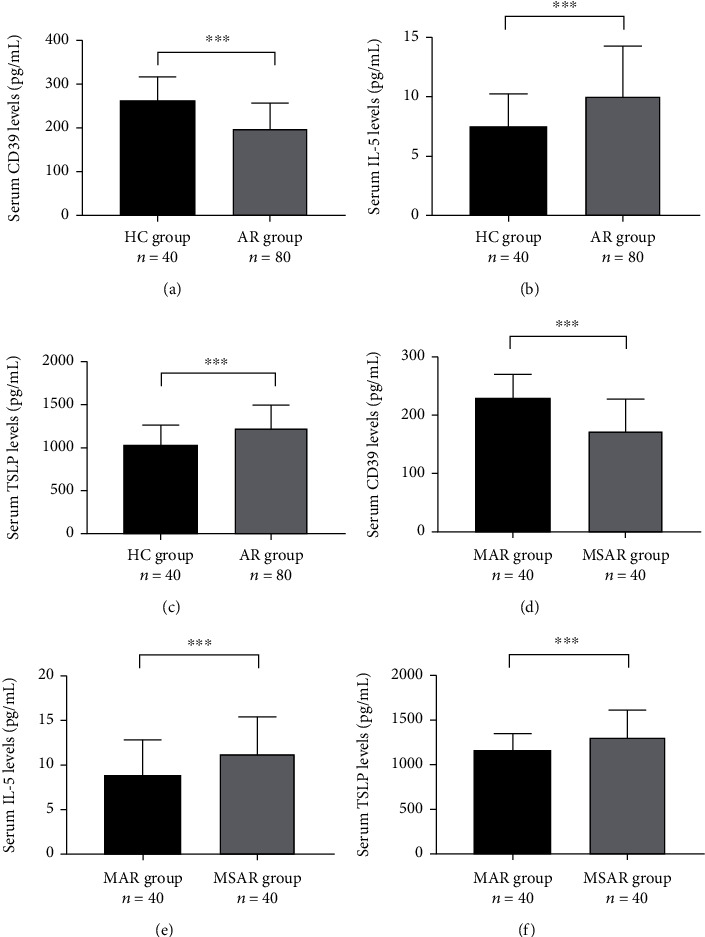
The serum levels of CD39, IL-5, and TSLP between AR patients and HCs in the validation cohort. IL: interleukin; TSLP: thymic stromal lymphopoietin; AR: allergic rhinitis; HC: healthy control.

**Figure 6:**
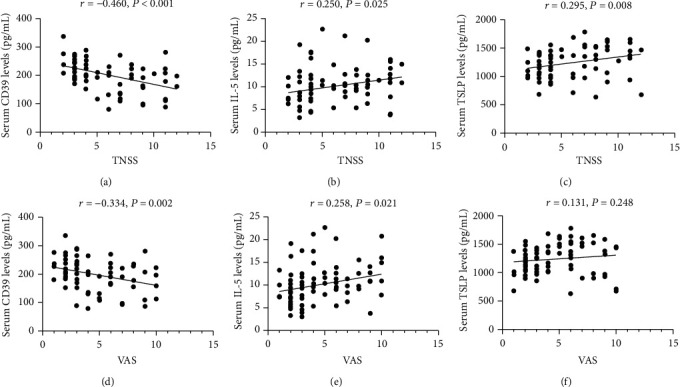
The associations between potential cytokines and TNSS and VAS in the validation cohort. TNSS: total nasal symptom score; VAS: visual analogue score.

**Figure 7:**
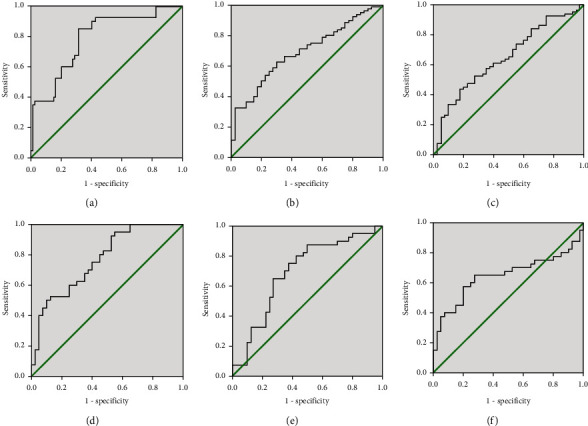
ROC curves of the capabilities of CD39, IL-5, and TSLP in diagnosing AR (a–c) and distinguishing disease severity (d–f). ROC: receiver operator characteristic; IL: interleukin; TSLP: thymic stromal lymphopoietin; AR: allergic rhinitis.

These cytokines with potential predictive abilities and differentially expressed between AR and HC groups, especially between MSAR and MAR groups, were verified by ELISA in the validation cohort.  Table 5 shows the demographic and clinical parameters of all participants among the three groups. The rates of accompanying asthma and allergic conjunctivitis were statistically significant among the three groups, and TNSS and VAS were significantly different between MSAR and MAR groups (*P* < 0.05). The ELISA results in Figure 5 highlighted that circulating CD39 levels were lower, and IL-5 and TSLP levels were higher in the AR group than in the HC group, especially in the MSAR group (*P* < 0.05). Spearman's correlation analysis results observed significant associations between concentrations of these biomarkers and TNSS and VAS (*P* < 0.05) (Figure 6). ROC curves suggested that serum CD39 exhibited more strong power in both diagnosing AR and distinguishing disease severity than the other two indicators (Figure 7). The detailed parameters are listed in Table S3 and Table S4.

## 4. Discussion

AR is an inflammation in the nose caused by complex etiologies, and its diagnosis and management are extremely challenging [15, 16]. AR can be grouped into MAR and MSAR based on clinical symptom scores; this method is subjective and relatively insensitive [4]. Although previous studies proposed multiple indicators to evaluate the disease severity in AR, including nasal nitric oxide, lipids, peripheral lymphocytes, and metabolites, none of them exhibited adequate sensitivity and specificity [6, 8, 17]. Therefore, exploring subjective biomarkers for AR diagnosis and reflecting disease severity remains a hot topic of significant research.

In order to address this issue, we conducted a discover–validation study with a combined application of peripheral multiple cytokine profile and ELISA to identify potential cytokines which were disease-specific and can be used in reflecting disease severity in AR patients. Our data demonstrated that discriminative serum cytokine profiles were observed between MSAR and MAR patients and HCs. Moreover, serum CD39, IL-5, and TSLP levels were proved to be associated with symptom scores and exhibited different capabilities in diagnosing AR and distinguishing its disease severity. The further validation results confirmed the diagnostic and predictive abilities of serum CD39, IL-5, and TSLP, and serum CD39 was proved to be more reliable than the other two cytokines.

CD39, also known as ectonucleotide triphosphate diphosphohydrolase-1 (E-NTPDase1), is the most prominent ATP hydrolyzing enzyme [18–20]. It was proven that extracellular ATP was a danger signal in innate immunity, and it triggered inflammasome activation and oxidative stress response and promoted the production of a series of proinflammatory cytokines, such as IL-1*β* and IL-8 [21–23]. CD39 could catalyze the conversion of extracellular ATP and ADP into AMP, promote the generation of adenosine, and be regarded as a critical immunoregulatory molecule which suppressed in the presence of inflammation [24–26]. Recent studies demonstrated that gene knockout of CD39 and CD73 gene knockout exacerbated allergic airway inflammation in mice via increasing the production of cytokines and recruitment of eosinophils [21, 27]. In another publication, Huang et al. reported that CD39 could alleviate airway hyperresponsiveness, eosinophilia, mucin deposition, and Th2 cytokine production, which was regarded as an essential regulator in airway inflammation [22]. In this study, we observed that serum CD39 levels were decreased in AR patients and reversely correlated with symptom scores, and serum CD39 exhibited powerful abilities in diagnosing AR and reflecting its disease activities. It is well known that Th2 and eosinophil inflammation are the predominated pathogenesis in AR, and Th2-related cytokines levels and the degree of eosinophil infiltration are considered to be associated with disease activity [28–30]. Accordingly, nucleotide- and nucleoside-mediated CD39 signaling was involved in the homeostatic regulation of eosinophil, elevated CD39 expression, inhibited eosinophil extravasation and accumulation, and alleviated the allergic symptoms in AR and asthma [31, 32]. Zhang et al. [33] found that allergen-induced extracellular ATP accumulation inhibited CD39 expression and eliminated the CD39-mediated suppression of IL-25, IL-33, and TSLP expression and group 2 innate lymphoid cell expansion. Therefore, we speculated that serum CD39 might be involved in the occurrence and development of AR; it could be a subjective biomarker for disease severity. However, its precise effect on the mechanisms of AR was not well clarified.

Our results also provided evidence that serum IL-5 and TSLP levels were elevated in AR patients and associated with disease severity, which was in accordance with most prior publications. IL-5 was a representative Th2 cytokine which was proven to be involved in the immunopathogenesis of AR [34, 35]. Previous studies demonstrated that circulating Th2 cytokines, including IL-4, IL-5, and IL-13, were upregulated in AR patients, and their concentrations roughly reflected the disease activity [36, 37]. TSLP, also known as an epithelium-derived proinflammatory cytokine, was demonstrated to be a master regulator of allergic airway inflammation and acted a pivotal role in the pathogenesis of allergic diseases, including AR [38–40]. Previous studies showed that enhanced TSLP levels aggravated the immune response toward a Th2 phenotype and promoted eosinophil infiltration and tissue remodeling in airway mucosa [41, 42]. Zheng et al. [43] found that TSLP and TSLP receptors were overexpressed in myeloid dendritic cells (DCs) and positively correlated with the degree of Th2-polarizing in AR patients. All the above events suggested that IL-5 and TSLP might be closely associated with the occurrence and development of AR, and they seemed to be promising biomarkers for monitoring the disease severity.

Several limitations exist in the present study, which may affect the clinical applications of our results. First, the number of participants was relatively small and recruited from a single medical center; results need to be validated in a larger multicenter cohort. Second, the disease severity is evaluated with TNSS and VAS, which are relatively subjective, and the associations between cytokine levels and disease severity seem to be not precise. Finally, other cytokines with no statistical difference or low predictive abilities are not further validated and discussed in this study, but it does not mean that they are not involved in AR.

## 5. Conclusion

In the present study, we observed significant differences in serum cytokine profile between AR patients and HCs and identified several cytokines associated with disease severity. The discover–validation cohorts suggested that serum CD39 exhibited strong abilities in diagnosing AR and reflecting its disease severity. These results provided new clues to improving the clinical management of AR through the exploration of more accurate diagnostics and disease-specific biomarkers.

## Figures and Tables

**Table 1:** Demographic and clinical parameters of HCs and AR patients.

Variables	HC group (*n* = 22)	AR group (*n* = 50)	*P* value
Gender			1.000
Male	11	26	
Female	11	24	
Age, years	30.6 ± 7.2	31.6 ± 7.5	0.620
BMI (kg/m^2^)	23.2 ± 1.1	23.1 ± 1.2	0.667
Accompanying diseases			
Asthma	0	15	0.003
Allergic conjunctivitis	0	7	0.092
TNSS	—	5.8 ± 3.0	NA
VAS	—	4.3 ± 2.5	NA

HC: healthy control; AR: allergic rhinitis; BMI: body mass index; TNSS: total nasal symptom score; VAS: visual analogue score; NA: not applicable.

**Table 2:** Demographic and clinical parameters of participants in MAR and MSAR groups.

Variables	MAR group (*n* = 25)	MSAR group (*n* = 25)	*P* value
Gender			1.000
Male	13	13	
Female	12	12	
Age, years	31.6 ± 7.6	31.6 ± 7.5	0.985
BMI (kg/m^2^)	23.1 ± 1.1	23.1 ± 1.3	0.925
Accompanying diseases			
Asthma	4	11	0.238
Allergic conjunctivitis	3	4	1.000
TNSS	3.3 ± 0.7	8.3 ± 2.4	<0.001
VAS	2.3 ± 0.7	6.3 ± 1.9	<0.001

MAR: mild allergic rhinitis; MSAR: moderate-severe; BMI: body mass index; TNSS: total nasal symptom score; VAS: visual analogue score.

**Table 3:** Cytokines levels in HC group and AR group (pg/mL).

Cytokines	HC group (*n* = 22)	AR group (*n* = 50)	*P* value
CD38	865.3 ± 155.5	857.7 ± 195.3	0.871
CD39	146.6 ± 57.0	78.4 ± 31.4	<0.001
CD73	4996.5 ± 659.5	4790.1 ± 1078.4	0.410
IFN-*γ*	7.8 ± 3.2	5.6 ± 2.4	0.003
IL-10	2.8 ± 3.1	2.7 ± 3.7	0.891
IL-13	1.8 ± 0.8	2.8 ± 2.0	0.026
IL-17A	6.6 ± 3.4	7.0 ± 2.9	0.567
IL-1*β*	2.3 ± 1.7	2.5 ± 0.9	0.601
IL-2	0.7 ± 0.6	0.7 ± 0.6	0.923
IL-25	336.2 ± 172.5	343.9 ± 124.7	0.832
IL-3	8.4 ± 2.9	8.3 ± 4.4	0.968
IL-33	222.7 ± 18.7	241.9 ± 37.3	0.025
IL-4	2.0 ± 0.5	1.9 ± 0.5	0.305
IL-5	1.9 ± 1.0	2.7 ± 1.6	0.030
IL-6	0.9 ± 0.8	0.9 ± 0.6	0.781
IL-7	4.3 ± 2.1	4.4 ± 2.4	0.889
IL-8	90.5 ± 43.6	91.6 ± 45.9	0.925
IL-9	231.6 ± 25.7	233.0 ± 28.1	0.840
TGF-*β*1	73.1 ± 16.8	72.3 ± 20.7	0.866
TGF-*β*2	19.2 ± 4.7	18.9 ± 7.2	0.858
TSLP	780.2 ± 306.1	942.5 ± 280.0	0.031

HC: healthy control; AR: allergic rhinitis; IFN: interferon; IL: interleukin; TGF: transforming growth factor; TSLP: thymic stromal lymphopoietin.

**Table 4:** Cytokines levels in MAR group and MSAR group.

Cytokines	MAR group (*n* = 25)	MSAR group (*n* = 25)	*P* value
CD38	882.5 ± 220.9	832.7 ± 166.7	0.372
CD39	91.6 ± 30.1	65.3 ± 26.8	<0.001
CD73	5000.4 ± 1150.1	4579.8 ± 979.6	0.170
IFN-*γ*	5.6 ± 2.9	5.7 ± 2.0	0.908
IL-10	3.8 ± 1.0	3.2 ± 2.6	0.045
IL-13	2.4 ± 0.8	2.8 ± 2.0	0.173
IL-17A	6.3 ± 2.4	7.7 ± 3.1	0.078
IL-1*β*	2.4 ± 1.0	2.6 ± 0.8	0.531
IL-2	0.8 ± 0.8	0.5 ± 0.4	0.201
IL-25	352.4 ± 132.4	335.4 ± 118.5	0.634
IL-3	7.8 ± 4.5	9.0 ± 4.2	0.288
IL-33	236.7 ± 31.4	247.2 ± 42.4	0.323
IL-4	1.9 ± 0.6	1.9 ± 0.4	0.775
IL-5	2.2 ± 0.6	3.1 ± 1.9	0.044
IL-6	0.9 ± 0.8	0.8 ± 0.5	0.556
IL-7	4.8 ± 2.6	3.9 ± 2.0	0.183
IL-8	84.9 ± 43.9	98.3 ± 47.7	0.308
IL-9	235.3 ± 20.5	230.7 ± 34.3	0.574
TGF-*β*1	67.5 ± 20.4	77.0 ± 20.2	0.104
TGF-*β*2	19.2 ± 8.0	18.6 ± 6.5	0.742
TSLP	861.8 ± 263.9	1023.1 ± 287.8	0.047

MAR: mild allergic rhinitis; MSAR: moderate-severe; IFN: interferon alpha; IL: interleukin; TGF: transforming growth factor; TSLP: thymic stromal lymphopoietin.

**Table 5:** Demographic and clinical parameters of participants in the validation cohort.

Variable	HC group (*n* = 40)	MAR group (*n* = 40)	MSAR group (*n* = 40)	*P* value
Gender				0.967
Male	20	20	21	
Female	20	20	19	
Age, years	31.8 ± 8.1	31.2 ± 7.3	32.0 ± 8.2	0.857
BMI, kg/m^2^	23.2 ± 1.3	23.1 ± 1.1	23.4 ± 1.7	0.6831
Accompanying diseases				
Asthma	0	7	10	0.004
Allergic conjunctivitis	0	4	6	0.047
TNSS	—	3.3 ± 0.7	8.5 ± 2.1	<0.001
VAS	—	2.3 ± 0.7	6.6 ± 2.0	<0.001

HC: healthy control; MAR: mild allergic rhinitis; MSAR: moderate-severe; BMI: body mass index; TNSS: total nasal symptom score; VAS: visual analogue score.

## Data Availability

The data utilized to support the observations of this study are available from the corresponding author upon request.

## Supplementary Materials

Supplementary Materials Table S1: ROC analysis results of different cytokines in diagnosing AR. Table S2: ROC analysis results of potential cytokines in distinguishing MSAR patients from MAR patients. Table S3: ROC analysis results of potential cytokines in diagnosing AR in the validation cohort. Table S4: ROC analysis results of potential cytokines in distinguishing MSAR from MAR in the validation cohort.
