# Supplementary material for: Peripheral Multiple Cytokine Profiles Identified CD39 as a Novel Biomarker for Diagnosis and Reflecting Disease Severity in Allergic Rhinitis Patients
Source: Mediators Inflamm. 2023 May 10;2023:3217261. doi: 10.1155/2023/3217261 (PMC10191753; doi:10.1155/2023/3217261)
Supplement: Supplementary Materials — Table S1: ROC analysis results of different cytokines in diagnosing AR. Table S2: ROC analysis results of potential cytokines in distinguishing MSAR patients from MAR patients. Table S3: ROC analysis results of potential cytokines in diagnosing AR in the validation cohort. Table S4: ROC analysis results of potential cytokines in distinguishing MSAR from MAR in the validation cohort. [file 3217261.f1.docx]

Table S1 ROC analysis results of different cytokines in diagnosing AR

| Cytokines | AUC (95%) | P value | cutoff value | sensitivity | specificity |
| --- | --- | --- | --- | --- | --- |
| CD39 | 0.830 (0.734-0.926) | <0.001 | 125.9 | 0.682 | 0.840 |
| IFN-γ | 0.630 (0.527-0.795) | 0.030 | 6.3 | 0.636 | 0.620 |
| IL-13 | 0.740 (0.618-0.862) | 0.001 | 1.9 | 0.700 | 0.682 |
| IL-33 | 0.701 (0.583-0.820) | 0.007 | 242.3 | 0.460 | 0.955 |
| IL-5 | 0.687 (0.544-0.831) | 0.012 | 1.3 | 0.940 | 0.409 |
| TSLP | 0.673 (0.528-0.818) | 0.020 | 670.1 | 0.880 | 0.455 |

ROC, receiver operator characteristic; AR, allergic rhinitis; AUC, area under the curve; CI, confidence interval; IFN, interferon alpha; IL, interleukin; TSLP, thymic stromal lymphopoietin.

Table S2 ROC analysis results of potential cytokines in distinguishing MSAR patients from MAR patients

| Cytokines | AUC (95%CI) | P value | cutoff value | sensitivity | specificity |
| --- | --- | --- | --- | --- | --- |
| CD39 | 0.891 (0.800-0.983) | <0.001 | 71.2 | 0.880 | 0.840 |
| IL-10 | 0.613 (0.455-0.771) | 0.171 | 3.4 | 0.280 | 0.960 |
| IL-5 | 0.662 (0.508-0.815) | 0.049 | 2.4 | 0.720 | 0.640 |
| TSLP | 0.704 (0.560-0.848) | 0.013 | 872.3 | 0.760 | 0.640 |

ROC, receiver operator characteristic; MAR, mild allergic rhinitis; MSAR, moderate-severe; AUC, area under the curve; CI, confidence interval; IL, interleukin; TSLP, thymic stromal lymphopoietin.

Table S3 ROC analysis results of potential cytokines in diagnosing AR in validation cohort

| Cytokines | AUC (95%CI) | P value | cutoff value | sensitivity | specificity |
| --- | --- | --- | --- | --- | --- |
| CD39 | 0.788 (0.703-0.874) | <0.001 | 28.5 | 0.880 | 0.840 |
| IL-5 | 0.690 (0.594-0.785) | 0.001 | 2.4 | 0.720 | 0.640 |
| TSLP | 0.647 (0.545-0.749) | 0.009 | 872.3 | 0.760 | 0.640 |

ROC, receiver operator characteristic; AR, allergic rhinitis; AUC, area under the curve; CI, confidence interval; IL, interleukin; TSLP, thymic stromal lymphopoietin.

Table S4 ROC analysis results of potential cytokines in distinguishing MSAR from MAR in validation cohort

| Cytokines | AUC (95%CI) | P value | cutoff value | sensitivity | specificity |
| --- | --- | --- | --- | --- | --- |
| CD39 | 0.768 (0.666-0.870) | <0.001 | 28.5 | 0.880 | 0.840 |
| IL-5 | 0.692 (0.573-0.810) | 0.003 | 2.4 | 0.720 | 0.640 |
| TSLP | 0.646 (0.519-0.774) | 0.024 | 872.3 | 0.760 | 0.640 |

ROC, receiver operator characteristic; MAR, mild allergic rhinitis; MSAR, moderate-severe; AUC, area under the curve; CI, confidence interval; IL, interleukin; TSLP, thymic stromal lymphopoietin.
